# Coronavirus disease: challenges for psychiatry

**DOI:** 10.1192/bjp.2020.86

**Published:** 2020-04-15

**Authors:** Brendan D. Kelly

**Affiliations:** Department of Psychiatry, Trinity College Dublin, Ireland

**Keywords:** COVID-19, coronavirus, low- and middle-income countries, emergency psychiatry, mental health service

## Abstract

Coronavirus disease (COVID-19) presents two urgent health problems: the illness caused by the virus itself and the anxiety, panic and psychological problems associated with the pandemic. Both problems present substantial challenges for our patients, their families, our multidisciplinary teams and our psychiatrist colleagues. We need good psychiatry, now more than ever.

In late December 2019, a patient in Wuhan Jinyintan Hospital in Hubei province in China was diagnosed with pneumonia.^1^ The illness was caused by a novel coronavirus disease, named COVID-19. By 20 February 2020, there were over 75 000 cases of COVID-19 in China. The median age of those infected was 51 years, but ranged from just 2 days to 100 years. Over three quarters of cases were aged between 30 and 69 years. Just over half were male.

COVID-19 was quick to spread beyond China's borders. On 11 March, the World Health Organization (WHO) declared a pandemic. By early April, the WHO reported that there were 1.25 million confirmed cases worldwide and over 69 000 deaths.

It is now clear that COVID-19 presents two major health problems. The first problem is the illness caused by the virus itself, which is usually self-limiting but can be fatal, especially in the vulnerable, the elderly and people with underlying health conditions. The second problem is the anxiety and panic that the virus triggers in the minds of virtually everyone who hears about it. Both problems present substantial challenges to psychiatry.

Regarding the first problem, the illness itself, much of the solution lies in the hands of public health authorities, the governments that fund them and citizens all around the world who adhere to public health advice about hand washing, coughing etiquette, not touching our faces, physical distancing and staying at home when advised to do so. These methods work to reduce community transmission of the virus.

In addition, medical staff around the globe are using established methods of infection control and treatment that were developed during previous outbreaks and are being adapted to this new challenge. Ultimately, COVID-19 will be controlled, albeit at very considerable cost to health, mental health and economies around the world.

The physical illness caused by COVID-19 is an urgent concern for psychiatry. We already know that people with mental illness have a lower life expectancy and poorer physical health outcomes than the general population.^2^ This means that our patients appear less likely to access public health advice about COVID-19, more likely to contract the virus and less likely to receive prompt diagnostic and treatment services.

Vulnerable populations are at particular risk: the homeless, people with disabilities, the chronically ill, prisoners and all who live in institutional settings. People in these groups often have accumulations of risk factors, including poor physical and mental health, impaired access to services and diminished control over their day-to-day lives.

As a result, there is a clear need for greater focus on providing care to the mentally ill and other vulnerable groups at this time, and a clear need to question any proposed re-deployment of mental health staff to other duties during the crisis, unless absolutely necessary.^3^ This is a mental health emergency as well as a physical health crisis.

One of the challenges with providing enhanced psychiatric care during the pandemic is the high risk of infection among psychiatrists and other mental health workers. Careful awareness of risk, judicious rostering and remote working where possible can help reduce this risk. This concern applies not only to fully trained psychiatrists and other mental health professionals, but also to trainees and students, whose education needs to progress as best as possible in the current, radically altered, circumstances. They, too, need our assistance. Fortunately, the website of the Royal College of Psychiatrists provides excellent information, advice and support (www.rcpsych.ac.uk). It is important that this continues and that we support the College's tireless work on COVID-19.

The second problem with the COVID-19 pandemic is the anxiety, panic and other mental health issues that are attributable to, or worsened by, COVID-19 among the public, health professionals, our patients, people with the infection and their families. Many of these problems are understandable in the context of a pandemic, but they still require careful consideration, accurate diagnosis and active management where necessary.

Among the public, free-floating anxiety can be helped by advising people to manage their use of media, especially social media, and focus on reliable sources of information, such as the WHO (www.who.int). This will help combat false information and conspiracy theories that simply stoke anxiety and cause enormous distress to many. Good diet, exercise and sleep are also important at times of heightened emotion, along with practices such as mindfulness and reaching out to others for mutual support.

Ironically, some of the public health measures required to control this infection can have negative effects on mental health. Quarantine, for example, can be associated with fear of infection, frustration, boredom and anxiety owing to lack of information.^4^ There can also be problems with stigma and finances afterwards. These problems can be mitigated by terminating these public health measures when they are no longer necessary, providing adequate information and basic supplies, reducing boredom and improving communication, using technology where possible. Children require particular attention to maintaining structure in their day and providing schooling as best as feasible. Children respond to honesty, practical suggestions (such as hand washing) and their parents setting a good example.

For people infected with COVID-19, there can be feelings of guilt, anxiety and despair, compounded by the physical effects of infection (cough, fever, hypoxia) and prolonged hospital stays. For the families of those affected, there can be feelings of guilt, remorse and loss. Following bereavement, grief is likely to be complex owing to limits on visiting the sick or dying in hospitals, uncertainties about the spread of infection and complications about the conduct of funerals. In this emotionally charged setting, psychological support is vital both at the time of death and into the future. The best strategy is to give people the time and space to rely on their informal networks to cope, but also ensure they know that the door is open to mental health services if they need them at any point, now or in the future.

Regrettably, there is already evidence that, in China at least, psychological and psychiatric care have been neglected in the context of COVID-19.^5^ Although this is partially understandable in the ‘white heat’ at the epicentre of a pandemic, it would be deeply unwise to neglect mental healthcare at this time. Never before has there been greater need for multidisciplinary mental health teams, clear communication about psychological supports, and use of new technologies to provide diagnostic and therapeutic services where and when they are needed.

As with virtually all outbreaks of infectious diseases, many of the people who die during the current pandemic will, in truth, die of a combination of COVID-19 and poverty. Although there is already considerable morbidity and mortality in high-income countries, these problems will inevitably be worse in countries with underdeveloped health systems, inadequate sanitation and poor nutrition. That is why, on 5 March 2020, Tedros Adhanom, Director-General of the WHO, emphasised the importance of global solidarity in addressing the pandemic:

‘Ultimately, how deadly this virus will be depends not only on the virus itself, but on how we respond to it. This is a serious disease. It is not deadly to most people, but it can kill. We're all responsible for reducing our own risk of infection, and if we're infected, for reducing our risk of infecting others. There's something all of us can do to protect vulnerable people in our communities. That's why we keep talking about solidarity. This is not just a threat for individual people, or individual countries. We're all in this together, and we can only save lives together.’^6^

For psychiatrists, this means solidarity with our patients, solidarity with their families, solidarity with our multidisciplinary colleagues and, perhaps most of all, solidarity with each other. We need each other now.

COVID-19 is the greatest public health challenge that most of us have ever encountered and, hopefully, it will be the worst that we ever encounter in the future. How psychiatry responds to this situation will play a large part in defining the nature and role of psychiatry in the years to come. The message from psychiatry's history is clear: we can rise to this challenge.

The core values of the Royal College of Psychiatrists are courage, innovation, respect, collaboration, learning and excellence. The COVID-19 pandemic requires that we translate all of these values into actions as a matter of urgency. COVID-19 demands no less.

Our patients need us now, more than ever.
